# Positive Selection Results in Frequent Reversible Amino Acid Replacements in the G Protein Gene of Human Respiratory Syncytial Virus

**DOI:** 10.1371/journal.ppat.1000254

**Published:** 2009-01-02

**Authors:** Viviane F. Botosso, Paolo M. de A. Zanotto, Mirthes Ueda, Eurico Arruda, Alfredo E. Gilio, Sandra E. Vieira, Klaus E. Stewien, Teresa C. T. Peret, Leda F. Jamal, Maria I. de M. C. Pardini, João R. R. Pinho, Eduardo Massad, Osvaldo A. Sant'Anna, Eddie C. Holmes, Edison L. Durigon, and the VGDN Consortium

**Affiliations:** 1 Butantan Institute, Virology Branch, Butantã, São Paulo, Brazil; 2 Laboratory of Molecular Evolution and Bioinformatics, Department of Microbiology, Institute of Biomedical Sciences, University of São Paulo, São Paulo, Brazil; 3 Division of Medical Biology, Adolfo Lutz Institute, São Paulo, Brazil; 4 Department of Cell Biology, School of Medicine of Ribeirão Preto, University of São Paulo, Ribeirão Preto, São Paulo, Brazil; 5 Pediatric Division, University Hospital of the University of São Paulo, São Paulo, Brazil; 6 Gastroenteritis and Respiratory Viruses Laboratory Branch, Division of Viral Diseases, National Center for Immunization and Respiratory Diseases, Coordinating Center for Infectious Diseases, Centers for Disease Control and Prevention, Atlanta, Georgia, United States of America; 7 STD/AIDS Reference and Training Centre, São Paulo, São Paulo, Brazil; 8 State University of São Paulo, São Paulo, Brazil; 9 Tropical Medicine Institute, University of São Paulo, São Paulo, Brazil; 10 Department of Legal Medicine, University of São Paulo Medical School, São Paulo, Brazil; 11 Center for Infectious Disease Dynamics, Department of Biology, The Pennsylvania State University, Mueller Laboratory, University Park, Pennsylvania, United States of America; 12 Fogarty International Center, National Institutes of Health, Bethesda, Maryland, United States of America; 13 Laboratory of Clinical Virology, Department of Microbiology, Institute of Biomedical Sciences, University of São Paulo, São Paulo, Brazil; Erasmus Medical Center, The Netherlands

## Abstract

Human respiratory syncytial virus (HRSV) is the major cause of lower respiratory tract infections in children under 5 years of age and the elderly, causing annual disease outbreaks during the fall and winter. Multiple lineages of the HRSVA and HRSVB serotypes co-circulate within a single outbreak and display a strongly temporal pattern of genetic variation, with a replacement of dominant genotypes occurring during consecutive years. In the present study we utilized phylogenetic methods to detect and map sites subject to adaptive evolution in the G protein of HRSVA and HRSVB. A total of 29 and 23 amino acid sites were found to be putatively positively selected in HRSVA and HRSVB, respectively. Several of these sites defined genotypes and lineages within genotypes in both groups, and correlated well with epitopes previously described in group A. Remarkably, 18 of these positively selected tended to revert in time to a previous codon state, producing a “flip-flop” phylogenetic pattern. Such frequent evolutionary reversals in HRSV are indicative of a combination of frequent positive selection, reflecting the changing immune status of the human population, and a limited repertoire of functionally viable amino acids at specific amino acid sites.

## Author Summary

As part of the Viral Genetic Diversity Network (VGDN), we sequenced the second variable region (G2) of the G protein of human respiratory syncytial virus (HRSV) A and B from 568 patients sampled during 11 consecutive HRSV seasons (1995–2005) in the state of São Paulo, Brazil. A total of 933 HRSVA and 673 HRSB time-stamped sequences, including those sampled here and globally, was used for phylogenetic inference and the analysis of selection pressures. We identified 18 positively selected sites in both HRSVA (9 sites) and HRSVB (9 sites) that tended to revert in time to their previous codon state (i.e. exhibited a “flip-flop” pattern). We argue that these common evolutionary reversals are indicative of frequent positive selection, reflecting the changing immune status of the human population, coupled with a limited repertoire of functional viable amino acids at specific sites. This information is of particular importance since the ectodomain of the G protein is also a target site in vaccines that have so far proven unsuccessful and because it constitutes a significant step towards describing and understanding the immune-escape repertoire of this major human pathogen.

## Introduction

Human respiratory syncytial virus (HRSV) is a leading cause of severe acute respiratory infection in childhood worldwide [1] and an important agent of acute respiratory infection in the elderly and immunocompromised [2],[3]. Initial studies with monoclonal antibodies to the HRSV F and G proteins divided the virus into two major groups (A and B) [4],[5]. Sequencing studies based on several HRSV genes have supported this major subdivision and lead to an additional genotypic classification, mainly based on the G protein gene, for epidemiological studies of HRSV. The genotypes of HRSVA and B show complex fluctuating dynamics, since they may co-circulate during a given season, with one or two dominant genotypes that are then replaced in consecutive years [6],[7],[8],[9],[10],[11].

The G protein is a target for neutralizing antibodies, interacts with host cell receptors and is highly variable [12],[13],[14],[15]. Most changes in the G protein are localized at an ectodomain containing two hyper-variable segments, separated by a highly conserved region between amino acids 164 and 176, assumed to represent a receptor-binding site [15]. Experimental data show that the G protein is not required for virus infection *in vitro* under appropriate conditions, but is necessary for efficient infection in mice and humans [16]. It has been argued that the antigenic variability of HRSV strains is one of the key features contributing to the ability of the virus to re-infect people and cause large-scale yearly outbreaks [17]. Moreover, several studies have shown that the C-terminal hyper-variable region of the surface G glycoprotein is immunogenic and contains multiple epitopes that are recognized by both murine monoclonal antibodies and human convalescent sera [18]. In addition, the deduced amino acid sequences of the G protein are highly divergent, with a sequence identity of approximately 53% between HRSVA and B, and 20% divergence within the same antigenic group [14],[19]. Despite this diversity, the nature of the selection pressures acting on the G protein have not been explored in detail, and particularly using sequence data sets that are of sufficient size to reveal the intricate nature of adaptive evolution and with restricted spatial and temporal sampling. This information is of particular importance since the ectodomain of the G-protein is also a target site in vaccines that have so far met with little success. To addresses these key issues we undertook the largest analysis of HRSV sequences undertaken to date, comprising both HRSVA and HRSVB, and utilizing detailed temporal information.

## Methods

### Respiratory samples

A total of 3,496 respiratory samples were used in this study. Nasopharyngeal aspirates and nasal swabs from 2,256 infants and young children (1 week to 5 years of age) hospitalized with acute respiratory lower infection (ARI) at University of São Paulo Hospital, São Paulo, Brazil were used. Samples were collected over 11 consecutive HRSV seasons (1995–2005). In addition, 1,240 respiratory samples from children with ARI were collected in 2004 and 2005 in different cities in São Paulo State and enrolled in the present study as part of the Viral Genetic Diversity Network (VGDN) (http://www.lemb.icb.usp.br/LEMB/index.php?p=11). Informed consent was obtained from parents or guardians of children enrolled in the study in the different cities according to a protocol approved by their respective Institutional Review Boards.

### HRSV screening

Specimens were collected in buffered saline and transported on ice to the laboratory for processing within 4 hours. A commercial immunofluorescence assay was used per manufacturer's instructions (Chemicon Light Diagnostics, Millipore Corp, Inc., Temecula, CA.), as previously described [20]. Clinical samples were amplified by RT-PCR as described bellow.

### RNA extraction and reverse transcription

Total RNA was extracted using guanidinium isothiocyanate phenol (Trizol LS, Invitrogen®, Carlsbad, CA) according to the manufacturer's instructions. Extracted RNA was annealed with 50 pmol random hexanucleotide primer (Invitrogen®) at 25°C for 25 minutes, followed by reverse transcription with 200 U SuperScript ™ (Invitrogen®) at 42°C for 1 hour.

### PCR and nucleotide sequencing

Partial HRSV G gene amplification was performed by a semi-nested PCR procedure. cDNA was amplified with reverse primer FV -5′GTTATGACACTGGTATACCAACC 3′ (based on sequences complementary to nucleotides 186 to 163 of the F protein gene messenger RNA strain CH18537 [21] – and the forward primer GAB - 5′YCAYTTTGAAGTGTTCAACTT 3′(G gene, 504–524 nt). A semi-nested PCR was then performed with primers F1AB -5′CAACTCCATTGTTATTTGCC3′ (F gene, 3–22 nt) and GAB [8],[9]. PCR assay was carried out in a reaction mixture containing 2,5 µL of cDNA, 20 mM Tris-HCl, 50 mM KCl, 1,5 mM MgCl_2_, 0,2 mM dNTPs, 10 pmol of each primer, 1,25 U of Taq DNA Polimerase (Taq-Gold, Applied Biosystems Inc) in a final volume of 25 µL. Amplification was performed in a GeneAmp PCR System 9700 thermocycler (Applied Biosystems Inc.) with the following parameters: 94°C for 5 minutes, followed by 35 cycles of 1 min at 94°C, 1 min at 55°C and 1 min at 72°C, and finally 7 min of extension at 72°C. The semi nested PCR was carried under the same conditions, with 10 pmol of each primer on a final volume of 25 µL. Both cDNA synthesis and PCR followed strict procedures to prevent contamination, including redundant negative controls and segregated environments for pre- and post-amplification procedures. Amplified products of the G gene showing the expected size by gel electrophoresis, were purified with a commercial kit (Concert Gel Extraction Systems, Invitrogen®), according to the manufacturer's instructions, followed by cycle-sequencing on a GeneAmp PCR System 9700 thermocycler (Applied Biosystems Inc). Sequence reactions were subjected to electrophoretic separation for primary data collection in ABI PRISM 3100 and 377 DNA sequencers (Applied Biosystems Inc.), using a fluorescent dye terminator kit (Applied Biosystems Inc.). Both strands were sequenced at least twice.

### Sequence editing and alignments

Sequences were assembled with the Sequence Navigator program version 1.0 (Applied Biosystems Inc., EUA) resulting in contigs of 270 nucleotides on average, corresponding to HRSV G gene nucleotide 649–918 (group A, prototype strain A2) and 652–921 (group B, prototype strain CH18537). Individual sequences were aligned to HRSV G references sampled globally [8],[9],[10],[11],[19],[22],[23],[24],[25],[26],[27],[28],[29],[30],[31],[32],[33],[34],[35],[36],[37] with the Se-Al - Sequence Alignment Editor [38], resulting in data sets with an average length of 270 nucleotides.

### Phylogenetic analyses

Because the strains A2 and Long (A group) and CH18537 and Sw8/60 (B group) were both the most divergent and considered prototype strains, they were included in the analysis as outgroup sequences for the phylogenetic analysis. The best–fit model of nucleotide substitution (GTR+Γ+I), and values for the shape parameter (α) for the distribution of among-site rate-heterogeneity distribution (Γ) were selected by hierarchical likelihood ratio testing using Modeltest Version 3.06 [39]. Using these models, maximum likelihood (ML) phylogenetic trees were inferred by heuristic searches using PAUP under sequentially the TBR (Tree Bisection-Reconnection), SPR (Subtree Pruning Regrafting) and NNI (Nearest Neighbor Interchange) perturbation procedures [40], using as BioNJ tree as a starting phylogeny. Levels of phylogenetic support for individual nodes were obtained by obtaining the majority rule consensus of the 100 best trees collected near the likelihood maxima during both the SPR and NNI branch-swapping procedures. Consensus values above 99% were used to check for genotype monophyly. Moreover, since samples had dates of sampling ranging a 30 year period, we also generated maximum clade credibility (MCC) trees for HRSVA and HRVB data using the Bayesian inference (BI) method in BEAST v. 1.4.7 [41]. We used the best fit model (GTR+Γ_4_+I ) assuming an uncorrelated lognormal-distributed relaxed clock with rates of change estimated from the data and using a Bayesian skyline demographic model as a coalescent prior. To obtain effective sampling sizes (ESS) above 100, MCC trees for HRSVA and HRSVB were obtained by pooling five independent Markov-chain Monte Carlo runs, each of which sampled from 10 million chains after a pre-burning period of 30 million chains.

### Analysis of selection pressures

To detect sites in the G protein that might be subject to positive selection we used the Bayesian methods implemented in the HyPhy program [42]. We employed the default ‘MG94xHKY85x3_4x2Rates with Rate heterogeneity, with 4 rate categories per parameter’ model. This estimates multiple parameters that are free to vary over sites both *d_N_* and *d_S_* to have distinct rates at a given site and to be sampled independently from two separate distributions. For the MG94xHKY85x3_4x2Rates model we used a Bayes factors >20 means that positive selection explains the data approximately 20 times better than the alternative model [42]. For comparison, we also used the less computationally intensive Single Likelihood Ancestral Counting (SLAC) and Fixed-Effects Likelihood (FEL) methods using the best fit nucleotide model estimated with HyPhy for each data set. With SLAC and FEL, all positively selected sites were estimated at the 95% confidence interval. We did not use the random effects likelihood method (REL) because of major computational constraints [42],[43],[44].

In addition, we obtained the most parsimonious reconstructions (MPR) of the positively selected sites along both HRSVA and HRSVB G protein trees using both the ‘accelerated transformation’ (ACCTRAN) method that maps character changes near the root of the tree, and the ‘delayed transformation’ (DELTRAN) method that maps character changes near the tips of the tree implemented in MacClade v. 4.07 [45]. Because all sequences had dates of sampling, allowing us to recover temporal patterns of amino acid replacement, we adjusted the tips of the phylogenies in time (*i.e.*, tip-dated trees) with BEAST v.1.4.7 (http://beast.bio.ed.ac.uk/).

### Nucleotide sequence accession numbers

The nucleotide sequences from the Brazilian isolates were deposited in the GenBank database under accession numbers EU582054 to EU582483, EU635778 to EU635865, EU259652 to EU259673, EU259675, EU259676, EU259678 to EU259690, EU259693 to EU259696 to EU259704, EU 625735, EU241632 to EU241634 and AY654589.

## Results

### HRSV sequencing and genotyping

We obtained nucleotide sequences of the second region (G2) of the HRSV G protein gene from (i) 432 random samples collected over 11 seasons (*i.e.*, from 1995 to 2005) from the city of São Paulo, Brazil and (ii) 136 sequences from samples collected by VGDN program from 2004 to 2005 from metropolitan area of the city of São Paulo and from the city of Ribeirão Preto, also in São Paulo state. Of a total of 568 sequences, 359 (63.2%) represented group A and 209 (36.8%) group B.

The Brazilian isolates of HSRVA had a deduced G protein of 298 or 297 amino acids, which was confirmed by complete G protein sequences obtained from several representative samples used in this study (data not shown). Interestingly, three isolates (Br89_2000, Br86_2000 and Br 206_2004) had a premature stop codon at amino acid position 288, which has been observed previously [46],[47] and four Brazilian GA5 strains from 2000 had a deletion of three bases, causing the loss of a serine residue at position 270. All the Brazilian isolates of HSRVB had an inferred G protein of 295 amino acids, except three isolates from 2000 season that had 299 amino acids due to a mutation in the first nucleotide of the stop codon of G gene, and two strains from 1999 season that had a Threonine codon insertion at position 233, leading to a G protein with 296 amino acids. Some strains isolated during 2001, 2003 and 2004 have an exact duplication of 60 nucleotides starting after residue 791 (accounting for a 20 aa duplication by insertion and resulting in a predicted protein of 312 to 319 aa). In 2005 this new genotype – denoted ‘GB3 with insertion’ (see below) – became the predominant. The alignment of partial amino acid sequences including the duplicated G segment showed some amino acid substitutions in the duplicate segment and in the 60 nucleotides immediately upstream. A total of 933 HRSVA sequences and 673 HRSVB sequences, including original and data compiled from GenBank, were used for further evolutionary analysis (see Table S1 in supplementary material).

### HRSV genotypes

**Figure 1:**
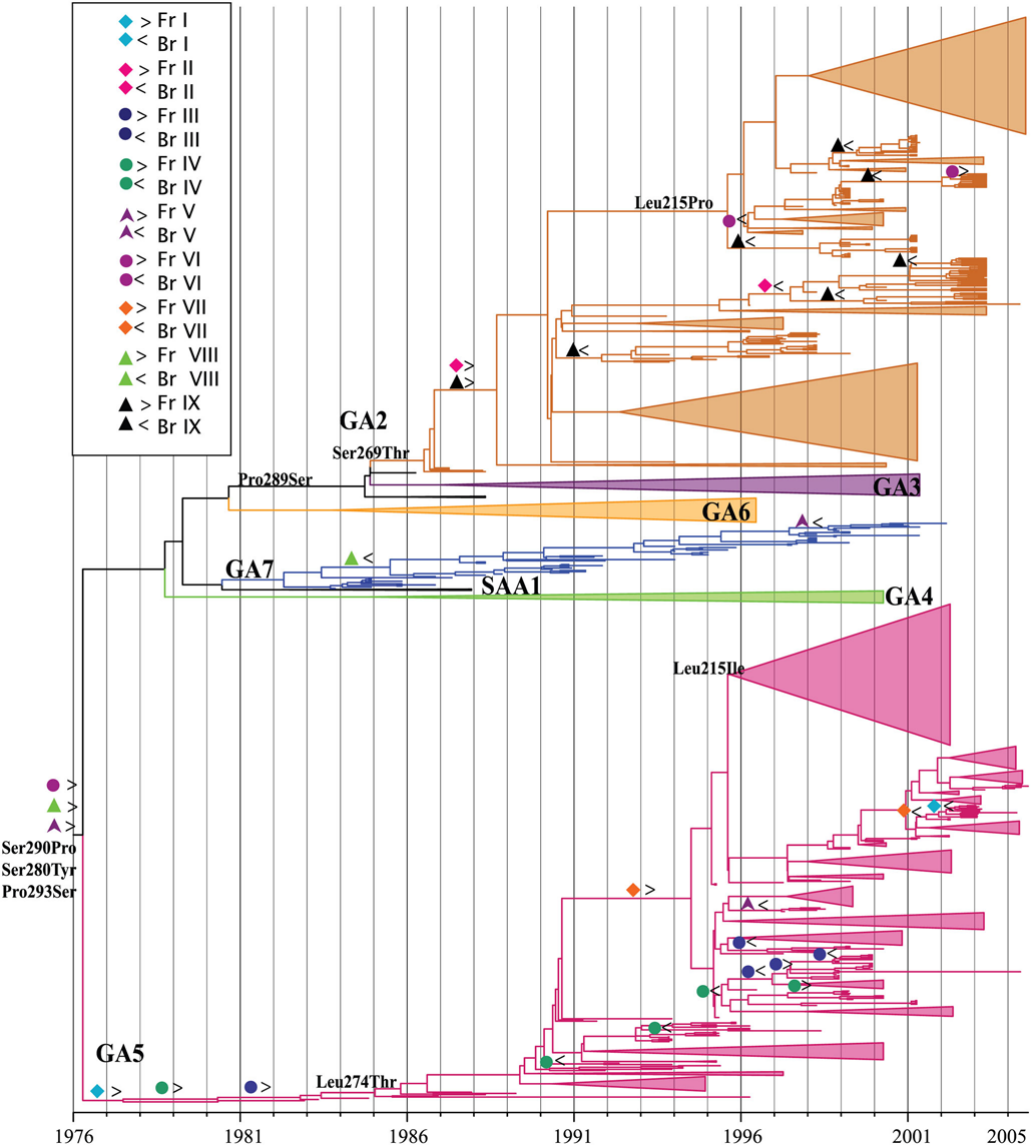
The most parsimonious reconstructions of positively selected sites on the HRSVA phylogeny rooted with the Long and A2 and GA1 strains (not shown for clarity) are depicted. Basal positively selected sites of HRSVA are indicated near the root of the tree. MPRs along the tree supporting the main splits are also indicated near the nodes at the base of each genotype. Lineages that did not experience evolutionary reversals were collapsed for the sake of clarity. For both A) and B) sites experiencing evolutionary mutations (Table 1) are indicated by the symbols: > for the ‘forward’ (Fr) mutation and < for the ‘backward’ mutation (Br). The Fr I is indicated by blue quadrilateral >, and the Br I by blue quadrilateral <. The Fr II is indicated by pink quadrilateral > and the Br II by pink quadrilateral <. The Fr III is indicated by blue circle > and the Br III by blue circle <. The Fr IV is indicated by green circle > and the Br IV by green circle <. The Fr V is indicated by violet triangle > and the Br V by violet triangle <. The Fr VI is indicated by violet circle > and the Br VI by violet circle <. The Fr VII is indicated by orange quadrilateral > and the Br VII by orange quadrilateral <. The Fr VII is indicated by green triangle > and the Br VIII by green triangle <. The Fr IX is indicated by black triangle > and the Br by black triangle <.

Both the ML and MCC trees divided HRSVA into seven monophyletic clusters with bootstrap or posterior probability support above 99%; these were previously described as genotypes GA1, GA2, GA3, GA4, GA6, GA5, GA7 and SAA1 [8],[9],[34]. The MCC tree (Fig. 1) showed that genotypes GA2, GA3, GA4, GA6 and GA7 had a common ancestor not shared by GA5 and GA1. GA2 strains fell into two distinct branches. One included the oldest strains isolated from 1995 to 2000 in several regions globally (*i.e.*, Brazil, South America, Belgian, United States and Africa). The other branch grouped the most recently isolated strains (2000 to 2004) that also exhibited a very widespread distribution (*i.e.*, Belgian, Brazil, China and Africa). Brazilian strains in this second branch were characterized by five amino acid substitutions: Leu215Pro, Arg244Lis, His266Tyr, Asp297Lys and the stop codon at 298 reverting to Trp (Stop298Trp). These changes were fixed in almost all 2003 to 2005 GA2 strains.

**Table 1:** Twenty-nine codon-sites under positive selection in the G gene of HRSVA including nine flip-flop sites (shown in Roman numerals) that were mapped in viral genealogy shown in Figure 1A, using three methods (Bayes factor >20 and *p*-value<0.05).

HRSVA						
Flip-flops§	aa position	Number of events	Change	MG94XHKY85	SLAC	FEL
	215 – Leu		Pro	*	*	*
	222 – Pro		Ser		*	
**I**	**225 – Val**					*
**FrI**	**Val:Ala**	1	Ala		*	
**BrI**	**Ala:Val**					
**II**	**226** –Pro			*	*	*
**Fr II**	**Pro:Leu**	1	Leu			
**Br II**	**Leu:Pro**					
	227 – Thr		Ala, Ile, Pro, Ser	*	*	
	230–Pro		Ser,Leu,Thr	*	*	
	237– Asn		His, Ser, Asp, Lys, Tyr	*	*	
**III**	**238 – Thr**			*	*	*
**Fr III**	**Thr:Ile**	4	Ile, Ala			
**Br III**	**Ile:Thr**					
	243 –Ile		Thr, Asn	*	*	*
	246 –Thr		Ile, Met	*	*	*
	248 –Leu		Phe, His, Val, Phe, Ile, Pro	*		
	249 – Thr		Ala, Ile, Asn, Ser	*	*	*
	253 –Thr		Ala,, Ile	*		
**IV**	**256** –Pro			*	*	*
**Fr IV**	**Pro:Leu**	4	Leu			
**Br IV**	**Leu:Pro**					
**V**	**265** –Phe			*		
**Fr V**	**Phe:Leu**	2	Leu			
**Br V**	**Leu:Phe**					
	269 –Ser		Thr	*		
**VI**	272 –Gly		Asp, Val, Ser	*	*	*
**Fr VI**	**274 – Leu**		Pro, Thr		*	
**Br VI**	**Leu:Pro**	2				
	**Pro:Leu**					
	275 –Ser		Gly, Ile, Asn, Arg	*	*	
**VII**	**279** – Val			*	*	
**Fr VII**	**Val:Ile**	1	Ile, Phe			
**Br VII**	**Ile:Val**					
	280 –Ser		Tyr	*	*	
	284 –Glu		Gly, Lys	*	*	*
	285 – Tyr or His		Asp, Phe, Asn, Ser	*	*	
**VIII**	**286 – Pro**		Leu		*	
**Fr VIII**	**Pro:Leu**	1				
**Br VIII**	**Leu:Pro**					
	287 – Ser		Leu, Pro	*	*	*
	289 –Pro		Ser	*	*	
**IX**	**290** – Ser			*		
**Fr IX**	**Pro:Leu**	6	Pro, Leu			
**Br IX**	**Leu:Pro**					
	292 – Pro		Thr, Ser	*	*	
	293 – Pro		Ser	*	*	

**§:** Fr- Forward replacements/Br – Backward replacements.

Sites detected by three methods are indicated by *.

**Figure 2:**
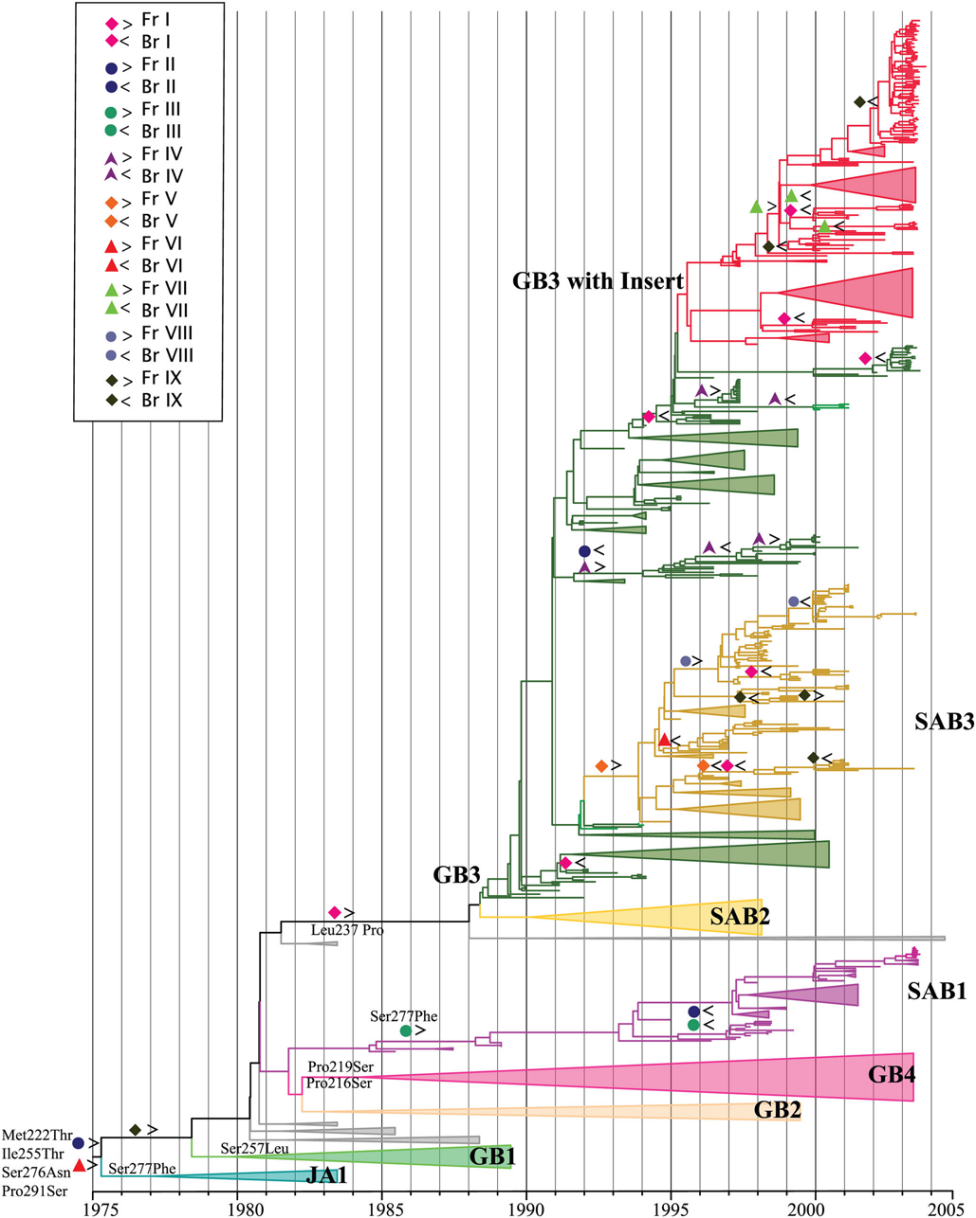
The most parsimonious reconstructions of positively selected sites on the HRSVA phylogeny rooted with the CH18537 and SW860 (not shown for clarity) are shown. Basal positively selected sites of HRSVB are indicated near the root of the tree. MPRs along the tree supporting the main splits are also indicated near the nodes at the base of each genotype. Lineages did not experience evolutionary reversals were collapsed for the sake of clarity. For both A) and B) sites experiencing evolutionary mutations (Table 1) are indicated by the symbols: pink quadrilateral > and the Br I by pink quadrilateral <. The Fr II is indicated by blue circle > and the Br by blue circle <. The Fr III is indicated by green circle > and the Br by green circle <. The Fr IV is indicated by violet triangle > and the Br by violet triangle<. The Fr V is indicated by orange quadrilateral > and the Br by orange quadrilateral <. The Fr VI is indicated by red triangle > and the Br by red triangle <The Fr VII is indicated by green triangle > and the Br VII by green triangle <. The Fr VIII is indicated by gray circle > and the Br VIII by gray circle <. The Fr IX is indicate by dark green quadrilateral > and the Br IX by dark green quadrilateral <.

The MCC tree for HRSVB (Fig. 2) contained 8 clusters that were previously described as genotypes JA1, GB1, GB2, GB3, GB4, SAB1, SAB2, SAB3 and GB3 with insertion [8],[9],[33],[34]. These same groupings were observed in the ML tree. GB3 was a paraphyletic genotype and included both SAB3 and the GB3 with the 60 nucleotide insertion.

### Selective Pressures in HRSV

In total, we found 29 sites to be subject to elevated rates of non-synonymous substitution (*d_N_*) in HRSVA, nine of which were detected to be under positive selection by the three methods we used and strongly suggesting that they are not false positives (Table 1). By using the Long 1956 prototype strain as an outgroup sequence, the most parsimonious reconstruction of the positively selected amino acid changes along the HRSVA tip-dated tree revealed that 22 mapped to the basal node (Fig. 1). That these 22 putatively positively selected sites included the replacement substitutions that defined the different genotypes and lineages within genotypes confirmed that they have reached a high frequency in the population again as expected of *bona fide* positively selected sites rather than false-positives. Site 215 had a Leucine (Leu) in all genotypes except in the non-circulating genotype GA1, that had a Proline (Pro), and the non-circulating Long 1956 prototype strain, that possessed a Histidine (His). Interestingly, most GA2 strains isolated after 2000 reverted to Pro at this site, and most GA5 strains isolate since 2001 changed to Isoleucine (Ile). Moreover, GA1 was basal to all other HRSVA genotypes and since it had no reversals on positive selected sites it was excluded from Fig. 1 for the sake of clarity (Fig. 1). The changes Phe265Leu, Leu274Pro, Ser280Tyr, Pro286Leu, Ser290Pro and Pro293Ser mapped to the split of two distinct branches; (*i*) one containing the older samples including prototypes (A2 and Long) and non-circulating genotype GA1 and, (*ii*) another containing the remaining genotypes (GA5, GA2, GA3, GA4, GA6 and GA7) (Fig. 1). Val225Ala, Pro256Leu, Thr238Leu and Leu274Thr changes defined the GA5 genotype (Fig. 1). Moreover, positively selected changes Pro289Ser, Pro226Leu, Ser269Thr and Pro290 Leu defined GA2 genotype, while Pro226Leu defined genotypes GA3 and GA7 (Fig. 1).

A total of 23 sites had elevated non-synonymous rates (*d_N_*) in HRSVB, thirteen of which were detected to be under positive selection by the three methods we used (Table 2). As with HRSVA, these sites defined lineages within genotypes, again suggesting that they are not false-positive results. The substitutions Pro216Ser and Pro219Ser defined the GB4 genotype, while changes Leu237Pro and Pro219Leu defined genotype GB3, and Thr255Ala defined SAB3 genotype. Moreover, site 277 changed from Ser to Phe in genotype JA1 (isolated in Japan) and in the SAB1 genotype. Finally, positively selected sites 242, 247, 255, 257 and 258 were located immediately upstream of the 20 amino acid-long duplication region while sites 267, 269 and 270 were located at the duplication region of the gene. The MPR of positively selected amino acid changes along the HRSVB tip-dated tree revealed that sites 222, 227, 255, 257, 276, 291 and 293 were likewise associated with the split of two distinct branches; (*i*) one containing the older samples including prototypes (CH18537 and Sw860) and non-circulating genotype JA1 and, (*ii*) other containing the remaining genotypes (SAB1, SAB2, SAB3 and GB3) (Fig. 2).

**Table 2:** Twenty-three codon-sites under positive selection in the G gene of HRSVB that were mapped in viral genealogy shown in Figure 1B, using three methods (Bayes factor >20 and *p*-value<0.05).

HRSVB						
Replacements§	aa position	Number of events	Changes	MG94XHKY85	SLAC	FEL
	216 – Pro		Ser	*	*	*
**I**	**219** – Pro			*	*	*
**Fr I**	**Pro:Leu**	7	Leu, Ser		*	*
**Br I**	**Leu:Pro**					
	222 - Met		Thr, Ala		*	*
	224 – Lys		Arg, Thr		*	*
**II**	**227** – Ile			*	*	*
**Fr II**	**Ile:Thr**	2	Thr		*	*
**Br II**	**Thr:Ile**					
	233 – Lys		Arg, Glu			*
**III**	**235** – Pro			*	*	*
**Fr III**	**Pro:Leu**	1	Leu			
**Br III**	**Leu:Pro**					
**IV**	**237** – Leu		Pro	*	*	*
**Fr IV**	**Pro:Ser**	3	Pro:Ser	*	*	*
**Br IV**	**Ser:Pro**					
	242 –Arg		Gly	*		
	246- Thr		Ile			*
	247 – Ser		Pro	*	*	*
**V**	**255** –Ile		Thr	*	*	*
**Fr V**	**Thr:Ala**	1	Thr:Ala			
**Br V**	**Ala:Thr**					
**VI**	**257** – Pro			*	*	*
**Fr VI**	**Pro:Ser**	1	Ser			
**Br VI**	**Ser:Pro**					
	258 – Lys		Asp, Asn, Gly		*	*
	269¶ – Ser		Leu, Pro, Phe	*	*	*
**VII**	**270** ¶ **– Thr**		Ile		*	*
**Fr VII**	**Thr:Ile**	2				
**Br VII**	**Ile:Thr**					
	285(265) – Ser		Phe, Pro, Thr, Tyr	*	*	*
**VIII**	**286 (266)** – Leu			*		*
**Fr VIII**	**Leu:Pro**	1	Pro			
**Br VIII**	**Pro:Leu**					
	297 (277) – Ser		Phe	*	*	*
	305 (285) –Glu		Asp, Lys	*		
	306 (286) –Pro		Leu	*		
	311 (291) –Pro		Ser	*	*	*
**IX**	**313 (293)** – Stop			*		*
**Fr IX**	**Stop: Gln**	5	Gln			
**Br IX**	**Gln :Stop**					

**¶:** Sites inside the 60 nt insertion. Positions between parentheses are position in sequences that do not have the 60 nt insertion. Sites 233, 246 and 275 were detected as being under elevated *dN*/*dS*, but show only apical changes in the tree and were excluded.

**§:** Fr- Forward replacements/Br – Backward replacements.

Sites detected by three methods are indicated by *.

**Figure 3:**
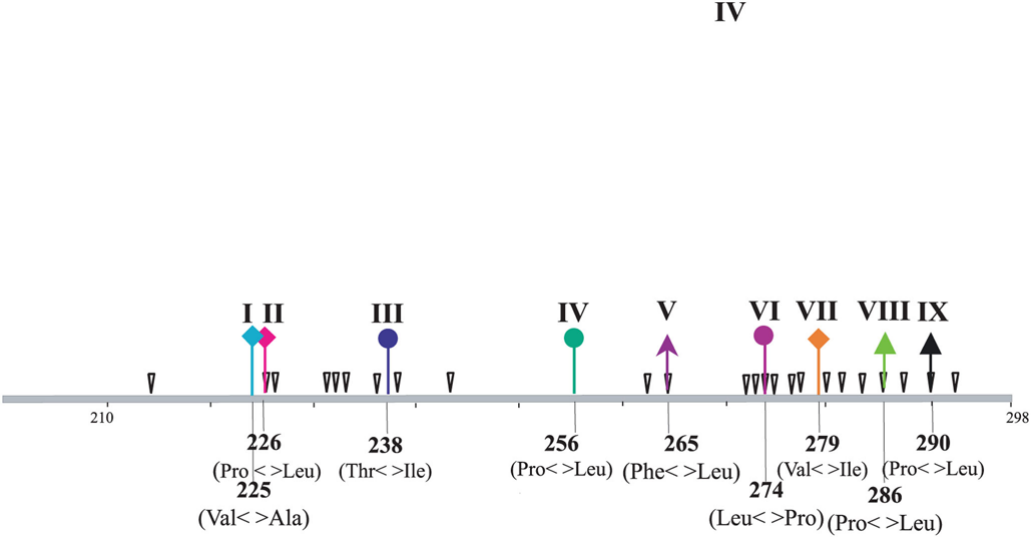
Graphical representation of the third C-terminal hypervariable region of HRSVA G [Long strain], showing a partial antigenic map. The amino acid changes associated with epitope loss in natural isolates and in escape mutants selected with specific Mabs are indicated by arrows [4],[15],[24],[40]. The positions, relative to the Long strain, of codons with evidence of positive selection that also experienced evolutionary reversals are shown by Roman numbers and coloured arrows.

**Figure 4:**
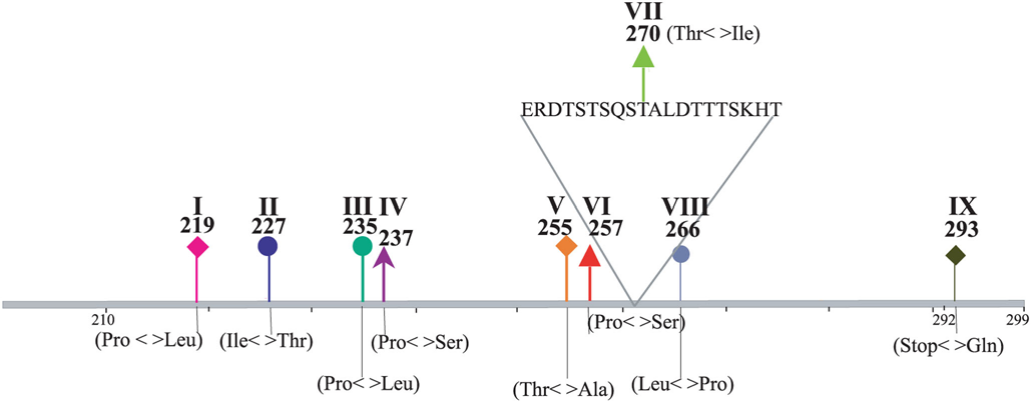
Graphical representation of the third C-terminal hypervariable region of HRSVB G (CH18537). The positions of the first amino acid in the amplicon and that for the insertion are shown in relation to the CH18537 isolate. The codon positions of sites with evidence of positive selective pressure that also experienced evolutionary reversals are indicated by Roman numbers and by coloured arrows.

However, perhaps the most notable observation of this analysis was that 18 of the total of the 55 putatively positively selected sites in HSRVA and B tended to revert, in time, to a previous codon state, indicative of a reversible (*i.e.*, “flip-flop”) pattern of amino acid replacement (shown in bold in Table 1 and 2, Fig. 1 and 2). Eleven of these 18 reversible sites in RSVA and RSVB were found to be positive selected under the most sensitive model (MG94xHKY85x3_4x2Rates model) but most by more than one models and by at least one model. Strikingly, such reversible evolution occurred at nine sites independently in *both* HRSVA and in HRSVB, although two sites in each virus group experienced reversal without detectable positive selection. For example, site 290 in GA2 genotype reverted from Leu to Pro five times along the tree (Table 1 and Fig. 1 and 3). Similarly, in some HRSV B genotypes (GB3 with insertion, GB3 and SAB3) site 219 reverted from Leu to Pro seven times along the tree (Table 2, Fig. 2 and 4).

## Discussion

The Brazilian isolates of G protein sequences of HRSV A and B demonstrated remarkable genetic flexibility, as noted previously at the global scale [33],[48],[49],[50],[51],[52]. Such a high level of genetic variation may be associated with the fact the G protein plays a key role in facilitating reinfections in HRSV – allowing evasion from cross-protective immune responses – and hence in the fluctuating patterns of viral circulation. As a consequence, describing the complex patterns of amino acid change in both HRSVA and HRSVB over time may help understand the evolution and epidemiology of this important virus.

Our analysis revealed that the ectodomain of the G protein was subject to strong positive selection, with 29 positively selected amino acid sites in HRSVA and 23 amino acid sites in HRSVB. The action of positive selection at these sites was also strongly supported since 18 of the 52 putatively positive selected sites were detected using all three forms of d_N_/d_S_ analysis. Only 5 of the 29 positively-selected sites in HRSVA have described previously (215, 225, 226, 256, 274 and 290) [10],[53],[54]. Possibly, this difference is due to the far larger data set available here and/or use of different analytical methods. Further, many of the positively selected sites in group A defined genotypes and lineages within genotypes, and correlated well with known epitopes described in escape-mutants selected with specific Mabs (sites 226, 237, 265, 274, 275, 284, 286 and 290) [25],[49],[51] or in natural isolates (sites 215, 225, 226, 265, 280, and 293) [13],[25],[46]. It is interesting to note that three of these sites (226, 265, 290) defined genotypes and underwent frequent reversals (Fig. 1). Site 237 was unique among positively selected sites in group A in that it had a residue – Asn – with the potential for N-glycosylation [14]. Moreover, six positively selected sites (225, 227, 253, 269, 275, 287) were previously described to have O-linked side chains [1]. The frequency and pattern of glycosylation were important in defining the antigenicity of the G protein, either by masking antigenic sites or by recognition of specific antibodies [55],[56]. Less is known about the effects of amino acid replacements at other sites (222, 227, 230, 243, 246, 248, 249, 272, 279, 285 and 292), although they were located close together to some of the epitopes involving in neutralizing the virus (Fig. 3). Moreover, we observed differences in the length of the G protein due to a stop codon mutation at site 298, and which was associated with the split of the tree in different branches. In GA5, Gln298 was maintained but changed to a stop codon (Gln298Stop) in both GA1 and the lineages leading to all other genotypes GA2, GA3, GA4, GA6 and GA7. Interestingly, the stop codon at 298 reverted to Trp in the GA2 branch that contains the most recent isolates. This reflects amino aid replacements involved in the presentation or elimination of multiple epitopes containing the three last residues of the G protein (*i.e.*, 296 to 298 C-terminal) [57].

Although epitopes in HRSVB are not well characterized, important differences in protein length between Brazilian strains were observed (295 or 299 amino acids), due to differences in the occurrence of the final stop codon (site 293). It was suggested that this region presents an epitope, substitutions in which would abolish the recognition of the G protein by strain specific antibodies [50],[54]. Moreover, the change at site 293 (stop codon:Gln) divided the tree in two distinct branches, one that included the ancient non-circulating strains and the other that included the recent strains. In almost all the Brazilian HRSV GB3 with insertion strains isolated in 2005 we observed an evolutionary “flip-flop” between a glutamine at site 293 and a stop codon, leading to a predicted G protein of 312 amino acid in length. Remarkably, other sites experienced similar reversals, such as amino acids 219, 227, 237 and 257, which defined new genotypes, suggesting that there are a limited number of amino acid residues at this site that allow successful virus attachment glycoprotein. Indeed, HRSV escape mutants that differ in their last 81 residues from the canonical Long prototype protein sequence, retain their compositions and hydropathy profiles [25], strongly suggesting that there may be indeed structural restrictions to changes in the G protein, although this will need to be investigated further. Finally, positively selected sites located in the 20 amino acid duplicated region of the gene, and immediately upstream of it may influence the expression of some important epitopes. For example, the additional O-linked glycosylation residues in both the insertion and duplication regions probably confers advantage of this new variant over the other HRSVB genotypes. Of the 23 positively selected sites in HRSVB, only five were described previously by Zlateva et al. 2005 (sites 219, 237, 247, 257 and 258) and, two by Woelk and Holmes, 2001 (sites 227 and 257). Consequently, HRSV appears to be subject to far greater positive selection pressure than previously realized.

Our data also identified amino acid sites under positive selection sharing positional homology in the two groups. For example, 11 positively selected sites in HRSVA (215, 226, 246, 256, 265, 274, 275, 284, 285, 290 and 292) had positional homologues in HRSVB (216, 227, 247, 257, 266, 275, 276, 285, 286, 291 and 293). Some of these sites are known to harbor epitopes in HRSVA (215, 226, 265, 275, 284 and 290). Moreover, some sites were important in defining lineages in the phylogenetic tree, such as sites 215, 265, 274, 286 and 290, specific to prototypes and non-circulating GA1 genotypes and site 226 defining the GA2 genotype. Less is known about epitopes in HRSVB, but sites 227, 257, 276, 291 and 293, under positive selection, were associated with the major division of the HRSVB phylogenetic tree into two branches.

The most interesting observation from this analysis was that both HRSVA and HRSVB experienced frequent evolutionary reversals of amino acids at positively-selected sites Tables 1 and 2, Figs. 1 and 2), which in turn mapped to known and possibly newly-described epitopes (Figs. 3 and 4). That most of the sites experiencing this “flip-flop” evolutionary pattern were also under positive selection strongly suggests that they reflect the fluctuating dynamics in the immune status of human populations, in which patterns of cross-protective immunity ebb and wane. To be more specific, the build-up of lineage-specific resistance in the host population would drive the process of positive selection in key immunological epitopes. Later, following the loss of herd immunity to the previous viral epitope, coupled with constraints which mean that only a limited number of amino acids are functionally viable, a reversion mutation would be fixed by positive selection in a newly susceptible human population. In sum, the frequent evolutionary reversals observed in the G protein of HRSV are a necessary consequence of a limited set of possible replacements at HRSV epitopes. Without such a constraint on the repertoire of functionally viable amino acids we would expect to see a gradual diversification at these sites rather than frequent reversals. This model agrees well with the spacing of temporal events observed in both viral phylogenies, supporting the notion that reversible evolution may contribute to the escape from the human population immune response, thereby facilitating viral transmission. A clearer understanding of the determinants of the evolutionary reversals within the G protein could ultimately lead to a better understanding of the viral immune-escape repertoire and assist in the control of HRSV.

## Supporting Information

Table S1 GeneBank Accession numbers
(0.67 MB DOC)
