# Supplementary material for: Positive Selection Results in Frequent Reversible Amino Acid Replacements in the G Protein Gene of Human Respiratory Syncytial Virus
Source: PLoS Pathog. 2009 Jan 2;5(1):e1000254. doi: 10.1371/journal.ppat.1000254 (PMC2603285; doi:10.1371/journal.ppat.1000254)
Supplement: Table S1 — GeneBank Accession numbers (0.67 MB DOC) [file ppat.1000254.s001.doc]

**Table S1.** GenBank Accession numbers of sequences used in this study.

| **HRSVA**  **Accession Number** | **HRSVA**  **Sample** | **HRSVB**  **Accession Number** | **HRSVB**  **Sample** |
| --- | --- | --- | --- |
| M11486 | A2 | M17213 | CH18537 |
| M17212 | Long | M55633 | Sw8/60 |
| AY226518 | G58S01 | AB175823 | NG-062-03 |
| AY226517 | G160S01 | AB175822 | NG-016-02 |
| AY226516 | G148S01 | AB175821 | NG-153-03 |
| AY226515 | 157KS01 | AB175820 | NG-006-03 |
| AY226514 | VG143S01 | AB175819 | NG-004-03 |
| AY226513 | 0240KS01 | AB161422 | T89 |
| AY226512 | 0128KS01 | AB161421 | S95-3 |
| AY226511 | 0069KS01 | AB161420 | S94-14 |
| AY146437 | Ab5076Pt01 | AB161419 | S93-2 |
| AY146436 | Ab4029B01 | AB161418 | T91-3 |
| AY146435 | Ab4026B01 | AB161417 | T85-2 |
| AY146434 | Ab3061CT01 | AB161416 | T93-3 |
| AY146433 | Ab3059CT01 | AB161415 | T91-2 |
| AY146432 | Ab164D00 | AB161414 | S02-71 |
| AY146431 | Ab105B00 | AB161413 | S02-29 |
| AY146430 | Ab83J00 | AB161412 | S01-31 |
| AY146429 | Ab47Pt00 | AB161411 | S01-22 |
| AY146428 | Ab86J00 | AB161410 | T98-4 |
| AY146427 | Ab84J00 | AB161409 | T98-3 |
| AY146426 | Ab81J00 | AB161408 | T98-2 |
| AY146425 | Ab104B00 | AB161407 | T99-3 |
| AY146424 | Ab55Pt00 | AB161406 | T97-4 |
| AY146423 | Ab34Pt00 | AB161405 | T97-3 |
| AY146422 | Ab11B00 | AB161404 | T97-2 |
| AY146421 | Ab31Ct00 | AB161403 | S88-24 |
| AY146420 | Ab21Bl00 | AB161402 | S87-18 |
| AY146419 | Ab54Pt00 | AB161401 | S86-288 |
| AY146418 | Ab43Ct00 | AB161400 | S87-245 |
| AY146417 | Ab24Ct00 | AB161399 | S85-99 |
| AY146416 | Ab65Pt00 | AB161398 | S85-94 |
| AY146415 | Ab20Bl00 | AB161397 | S98-1 |
| AY146414 | Ab56Pt00 | AB161396 | S85-31 |
| AY146413 | Ab39Ct00 | AB161395 | S85-17 |
| AY146412 | Ab71D00 | AB161394 | S84-497 |
| AY146411 | Ab78D00 | AB161393 | S84-455 |
| AY548802 | Ag32-00 | AB161392 | S84-369 |
| AY548801 | Ag-A48-99 | AB161391 | S84-352 |
| AY548800 | AgJ6-98 | AB161390 | S82-70 |
| AY548799 | AgK23-00 | AB161389 | S82-15 |
| AY548798 | AgK28-00 | AB161388 | S81-a |
| AY548797 | Ag38-00 | AB161387 | S80-732 |
| AF348810 | SA98D707 | AB161386 | S80-682 |
| AF348809 | SA97D669 | AB161385 | S92-10 |
| AF348808 | SA99V1239 | AB161384 | S91-6 |
| AF348807 | SA98V603 | AB161383 | S91-46 |
| AF348806 | SA98V173 | AB161382 | S91-30 |
| AF348805 | SA0003 | AB161381 | S90-157 |
| AF348804 | SA99V360 | AB161380 | S89-9 |
| AF348803 | SA97D1289 | AB161379 | S89-42 |
| AF348802 | SA97D804 | AB161378 | S98-30 |
| AY343659 | BE-369-90 | AB161377 | S98-25 |
| AY343659 | BE-191-90 | AJ290224 | UK70596/02/96 |
| AY343658 | BE-138-90 | AJ290222 | UK70248/02/96 |
| AY343657 | BE-8078-92 | AJ290219 | UK70053/02/96 |
| AY343656 | BE-119-87 | AJ290216 | UK70833/01/96 |
| AY343655 | BE-307-87 | AJ290215 | UK70705/01/96 |
| AY343654 | BE-3785-87 | AJ290213 | UK70107/01/96 |
| AY343653 | BE-305-89 | AJ290211 | UK70024/01/96 |
| AY343652 | BE-6460-91 | AJ290210 | UK70003/01/96 |
| AY343651 | BE-1440-92 | AJ290209 | UK70754/01/96 |
| AY343650 | BE-64-92 | AJ290207 | UK71073/12/95 |
| AY343649 | BE-6274-91 | AJ290206 | UK70901/12/95 |
| AY343648 | BE-6374-91 | AJ290205 | UK70870/12/95 |
| AY343647 | BE-12243-96 | AJ290204 | UK70739/12/95 |
| AY343646 | BE-13412-99 | AJ290203 | UK70454/01/96 |
| AY343645 | BE-11584-01 | AJ290202 | UK70760/12/95 |
| AY343644 | BE-11451-00 | AJ290201 | UK70642/12/95 |
| AY343643 | BE-13462-99 | AJ290200 | UK70545/12/95 |
| AY343642 | BE-1564-00 | AJ290199 | UK70457/12/95 |
| AY343641 | BE-3252-86 | AJ290198 | UK70319/12/95 |
| AY343640 | BE-410-86 | AJ290197 | UK70207/11/95 |
| AY343639 | BE-3933-87 | AJ290196 | UK70663/11/95 |
| AY343638 | BE-523-86 | AJ290214 | UK71207/01/96 |
| AY343637 | BE-3729-86 | AY660684 | Ken/2/03 |
| AY343636 | BE-3021-86 | AY660683 | Ken/23/03 |
| AY343635 | BE-764-86 | AY660682 | Ken/12/03 |
| AY343634 | BE-2140-84 | AY660681 | Ken/29/03 |
| AY343633 | BE-339-86 | AY660680 | Ken/253/02 |
| AY343632 | BE-76-86 | AY524578 | Ken/9/00 |
| AY343631 | BE-2466-85 | AY524577 | Ken/1/00 |
| AY343630 | BE-2052-85 | AY660576 | Ken/4/00 |
| AY343629 | BE-3833-88 | AY524575 | Ken/2/00 |
| AY343628 | BE-4763-88 | AY524574 | Ken/1/99 |
| AY343627 | BE-115-89 | AY524573 | Ken/109/02 |
| AY343626 | BE-183-85 | AY773291 | Ken/259/00 |
| AY343625 | BE-204-84 | AY773292 | Ken/13/03 |
| AY343624 | BE-538-88 | AY472098 | Sal/122-99 |
| AY343623 | BE-71-88 | AY472101 | sal/83/99 |
| AY343622 | BE-4147-87 | AY472100 | sal/141/99 |
| AY343621 | BE-512-95 | AY472099 | sal/84/99 |
| AY343620 | BE-1591-90 | AB117522 | S0004 |
| AY343619 | BE-12895-95 | AY226537 | VG72S01 |
| AY343618 | BE-13551-95 | AY226536 | V2004K01 |
| AY343617 | BE-15752-97 | AY226535 | V0041KS01 |
| AY343616 | BE-15739-97 | AY226534 | G36S01 |
| AY343615 | BE-11465-94 | AY226533 | 193KS01 |
| AY343614 | BE-14461-98 | AY226532 | 1802737S01 |
| AY343613 | BE-332-02 | AY226531 | 0227KS01 |
| AY343612 | BE-13393-99 | AY226530 | 0219KS01 |
| AY343611 | BE-11-01 | AY226529 | 0216KS01 |
| AY343610 | BE-14898-98 | AY226528 | 0140KS01 |
| AY343609 | BE-14808-98 | AY226527 | 0137KS01 |
| AY343608 | BE-12028-00 | AY226526 | 0112KS01 |
| AY343607 | BE-11996-00 | AY226525 | 0072S01 |
| AY343606 | BE-4-02 | AY226524 | 0064S01 |
| AY343605 | BE-519-01 | AY226523 | G31S01 |
| AY343604 | BE-1957-00 | AY226522 | G19S01 |
| AY343603 | BE-13425-99 | AY226521 | G94S01 |
| AY343602 | BE-21-00 | AY226520 | G7S01 |
| AY343601 | BE-11091-00 | AY226519 | G102S1 |
| AY343600 | BE-11129-00 | AY146444 | Ab3062C01 |
| AY343599 | BE-11030-00 | AY146443 | Ab3064C01 |
| AY343598 | BE-12023-00 | AY146442 | Ab5075P01 |
| AY343597 | BE-156-84 | AY146441 | Ab5078P01 |
| AY343596 | BE-2584-85 | AY146440 | Ab27CT00 |
| AY343595 | BE-933-88 | AY146439 | Ab41CT00 |
| AY343594 | BE-15471-97 | AY146438 | Ab17BL00 |
| AY343593 | BE-12350-96 | AF348826 | SA98D1656 |
| AY343592 | BE-12216-96 | AF348825 | SA0025 |
| AY343591 | BE-174-95 | AF348824 | SA98V602 |
| AY343590 | BE-12005-94 | AF348823 | SA99V439 |
| AY343589 | BE-12061-94 | AF348822 | SA99V1325 |
| AY343588 | BE-11600-94 | AF348821 | SA99V800 |
| AY343587 | BE-1587-89 | AF348820 | SA98V220 |
| AY343586 | BE-614-93 | AF348819 | SA98D941 |
| AY343585 | BE-462-94 | AF348818 | SA98V268 |
| AY343584 | BE-10490-93 | AF348817 | SA97D934 |
| AY343583 | BE-14536-98 | AF348816 | SA98D661 |
| AY343582 | BE-944-00 | AF348815 | SA98V153 |
| AY343581 | BE-797-00 | AF348814 | SA99V470 |
| AY343580 | BE-1345-00 | AF348813 | SA99V429 |
| AY343579 | BE-1150-00 | AF348812 | SA0028 |
| AY343578 | BE-112-01 | AF348811 | SA98V192 |
| AY343577 | BE-1717-01 | AY548809 | AgJ15-99 |
| AY343576 | BE-1836-01 | AY548808 | AgA23-98 |
| AY343575 | BE-1866-01 | AY548807 | AgJ16-99 |
| AY343574 | BE-1343-01 | AY548806 | AgK6-99 |
| AY343573 | BE-2149-00 | AY548805 | AgC13-00 |
| AY343572 | BE-1937-00 | AY548804 | AgA88-00 |
| AY343571 | BE-1224-01 | AY548803 | Ag21-00 |
| AY343570 | BE-1835-01 | M73540 | WV4843 |
| AY343569 | BE-1936-00 | AF065250 | CH10b |
| AY343568 | BE-13281-99 | AF065251 | CH93-9b |
| AY343567 | BE-822-00 | AF065252 | CH93-18b |
| AY343566 | BE-64-01 | AF065253 | CH93-53b |
| AY343565 | BE-11976-00 | AF233933 | TX69208 |
| AY343564 | BE-936-01 | AF233932 | NY97 |
| AY343563 | BE-901-01 | AF233931 | NY01 |
| AY343562 | BE-1441-01 | AF233930 | MO53 |
| AY343561 | BE-800-00 | AF233929 | MO35 |
| AY343560 | BE-1556-01 | AF233928 | MO30 |
| AY343559 | BE-2122-00 | AF233927 | CN3521 |
| AY343558 | BE-16-00 | AF233926 | CN1839 |
| AY343557 | BE-1061-00 | AF233925 | AL19794-1 |
| AY343556 | BE-13192-99 | AF233924 | AL19734-4 |
| AY343555 | BE-13172-99 | M73543 | NM1355 |
| AY343554 | BE-13280-99 | AF193338 | SK95100 |
| AY343553 | BE-1682-00 | AF193337 | SK95043 |
| AY343552 | BE-1834-00 | AF193336 | SK94212 |
| AY343551 | BE-13083-99 | AF193335 | SK94055 |
| AY343550 | BE-1617-00 | AF193334 | SK93418 |
| AY343549 | BE-11101-00 | AY751282 | BE/2756/85 |
| Z33421 | Mon1-87 | AY751281 | BE/1932/84 |
| Z33424 | Mon/2/88 | AY751280 | BE/395/85 |
| Z33425 | Mon/3/88 | AY751279 | BE/4183/87 |
| Z33422 | Mon1-89 | AY751278 | BE/2105/84 |
| Z33426 | Mon/4/90 | AY751277 | BE/2148/84 |
| Z33427 | Mon-5-90 | AY751276 | BE/2003/84 |
| Z33428 | Mon-5-91 | AY751275 | BE/400/91 |
| Z33431 | Mon-9-91 | AY751274 | BE/45/91 |
| Z33429 | Mon-7-91 | AY751273 | BE/66/91 |
| Z33432 | Mon-9-92 | AY751272 | BE/12308/96 |
| Z33430 | Mon/8/92 | AY751271 | BE/12160/02 |
| Z33423 | Mon-1-92 | AY751270 | BE/12031/99 |
| Z33412 | Mad-1-89 | AY751269 | BE/975/02 |
| Z33415 | Mad-3-89 | AY751268 | BE/11754/00 |
| Z33416 | Mad-4-90 | AY751267 | BE/10760/00 |
| Z33420 | MAd491 | AY751266 | BE/10557/00 |
| Z33418 | Mad-6-92 | AY751265 | BE/12024/00 |
| Z33417 | Mad/5/92 | AY751264 | BE/860/90 |
| Z33454 | Mad-6-93 | AY751263 | BE/5339/90 |
| Z33414 | Mad-1-93 | AY751262 | BE/450/83 |
| Z33411 | Mad-3-92 | AY751261 | BE/1733/82 |
| Z33410 | Mad-6-93 | AY751260 | BE/1732/82 |
| Z33419 | Mad-8-92 | AY751259 | BE/3455/86 |
| AF516137 | Mon/3/01 | AY751258 | BE/946/87 |
| AF516136 | Mon/2/01 | AY751257 | BE/2968/85 |
| AF516135 | Mon/1/01 | AY751256 | BE/1176/88 |
| AF516134 | Mon/7/00 | AY751255 | BE/4618/88 |
| AF516133 | Mon/6/00 | AY751254 | BE/391/89 |
| AF516132 | Mon/5/00 | AY751253 | BE/4383/89 |
| AF516131 | Mon/4/00 | AY751252 | BE/456/87 |
| AF516130 | Mon/3/00 | AY751251 | BE/3280/87 |
| AF516129 | Mon/2/00 | AY751250 | BE/4929/88 |
| AF516128 | Mon/1/00 | AY751249 | BE/5222/98 |
| AF516127 | Ar/2/98 | AY751248 | BE/128/92 |
| AF516126 | Ar/1/98 | AY751247 | BE/90/92 |
| AF516125 | Mon/2/98 | AY751246 | BE/12228/98 |
| AF516124 | Mon/1/98 | AY751245 | BE/14364/98 |
| AF516123 | Ar/3/97 | AY751244 | BE/11712/93 |
| AF516122 | Ar/2/97 | AY751243 | BE/7374/92 |
| AF516121 | Ar/1/97 | AY751242 | BE/283/00 |
| AF516120 | Mon/4/97 | AY751241 | BE/12252/96 |
| AF516119 | Mon/3/97 | AY751240 | BE/12624/96 |
| AF516118 | Mon/2/97 | AY751239 | BE/11900/96 |
| AF516117 | Mon/1/97 | AY751238 | BE/2098/00 |
| AF516116 | Ar/2/96 | AY751237 | BE/618/00 |
| AF516115 | Ar/1/96 | AY751236 | BE/522/93 |
| AF516114 | Mon/5/96 | AY751235 | BE/361/94 |
| AF516113 | Mon/4/96 | AY751234 | BE/949/94 |
| AF516112 | Mon/3/96 | AY751233 | BE/143/94 |
| AF516111 | Mon/2/96 | AY751232 | BE/215/94 |
| AF516110 | Mon/1/96 | AY751231 | BE/15/94 |
| AF516109 | Mon/2/95 | AY751230 | BE/11120/93 |
| AF516108 | Mon/1/95 | AY751229 | BE/1587/96 |
| AF516107 | Mon/1/93 | AY751228 | BE/612/96 |
| AF448498 | Mon/1/94 | AY751227 | BE/14273/95 |
| AY667075 | Mon/1/1990 | AY751226 | BE/11972/96 |
| AY660679 | Ken/4/03 | AY751225 | BE/12186/96 |
| AY660678 | Ken/16/03 | AY751224 | BE/12340/96 |
| AY660677 | Ken/5/03 | AY751223 | BE/12059/99 |
| AY660676 | Ken/19/03 | AY751222 | BE/1091/96 |
| AY660675 | Ken/260/02 | AY751221 | BE/13236/95 |
| AY660674 | Ken/44/03 | AY751220 | BE/376/96 |
| AY660673 | Ken/43/03 | AY751219 | BE/96/96 |
| AY660672 | Ken/262/02 | AY751218 | BE/1334/96 |
| AY660671 | Ken/56/03 | AY751217 | BE/154/91 |
| AY660670 | Ken/58/03 | AY751216 | BE/23/91 |
| AY660669 | Ken/73/03 | AY751215 | BE/10495/93 |
| AY660668 | Ken/79/03 | AY751214 | BE/11713/93 |
| AY660667 | Ken/81/03 | AY751213 | BE/12562/99 |
| AY524663 | Ken/7/00 | AY751212 | BE/196/01 |
| AY524662 | Ken/96/02 | AY751211 | BE/381/01 |
| AY524661 | Ken/9/02 | AY751210 | BE/13182/99 |
| AY524660 | Ken/9/01 | AY751209 | BE/381/00 |
| AY524659 | Ken/8/02 | AY751208 | BE/352/00 |
| AY524658 | Ken/8/01 | AY751207 | BE/13350/99 |
| AY524657 | Ken/8/00 | AY751206 | BE/11842/01 |
| AY524656 | Ken/73/02 | AY751205 | BE/12332/01 |
| AY524655 | Ken/7/02 | AY751204 | BE/8/02 |
| AY524654 | Ken/7/01 | AY751203 | BE/135/02 |
| AY524653 | Ken/69/02 | AY751202 | BE/11821/01 |
| AY524652 | Ken/66/02 | AY751201 | BE/11583/01 |
| AY524651 | Ken/61/02 | AY751200 | BE/11823/01 |
| AY524650 | Ken/6/02 | AY751199 | BE/11790/01 |
| AY660649 | Ken/6/00 | AY751198 | BE/13768/97 |
| AY660648 | Ken/57/02 | AY751197 | BE/11718/96 |
| AY660647 | Ken/55/02 | AY751196 | BE/11875/01 |
| AY524646 | Ken/54/02 | AY751195 | BE/277/96 |
| AY524645 | Ken/5/02 | AY751194 | BE/1542/98 |
| AY524644 | Ken/5/01 | AY751192 | BE/14516/97 |
| AY524643 | Ken/5/00 | AY751191 | BE/2803/98 |
| AY524642 | Ken/49/02 | AY751190 | BE/1509/96 |
| AY524641 | Ken/43/02 | AY751189 | BE/1845/96 |
| AY524640 | Ken/40/02 | AY751188 | BE/1959/96 |
| AY524639 | Ken/4/02 | AY751187 | BE/259/96 |
| AY524638 | Ken/4/01 | AY751186 | BE/3837/02 |
| AY524637 | Ken/3/02 | AY751185 | BE/12350/02 |
| AY524636 | Ken/3/01 | AY751184 | BE/2125/03 |
| AY524635 | Ken/3/00 | AY751183 | BE/1803/03 |
| AY524634 | Ken/28/02 | AY751182 | BE/12357/98 |
| AY524633 | Ken/27/02 | AY751181 | BE/14361/98 |
| AY524632 | Ken/26/02 | AY751180 | BE/705/01 |
| AY524631 | Ken/25/02 | AY751179 | BE/858/03 |
| AY524630 | Ken/24/02 | AY751178 | BE/13354/02 |
| AY660629 | Ken/231/02 | AY751177 | BE/12356/02 |
| AY524628 | Ken/23/02 | AY751176 | BE/922/03 |
| AY524627 | Ken/22/02 | AY751175 | BE/12237/02 |
| AY524626 | Ken/216/02 | AY751174 | BE/13415/02 |
| AY524625 | Ken/211/02 | AY751173 | BE/552/03 |
| AY524624 | Ken/21/02 | AY751172 | BE/13418/02 |
| AY524623 | Ken/209/02 | AY751171 | BE/756/03 |
| AY524622 | Ken/208/02 | AY751170 | BE/564/03 |
| AY524621 | Ken/204/02 | AY751169 | BE/600/03 |
| AY524620 | Ken/201/02 | AY751168 | BE/654/03 |
| AY524619 | Ken/20/02 | AY751167 | BE/369/03 |
| AY524618 | Ken/2/02 | AY751165 | BE/13491/02 |
| AY524617 | Ken/2/01 | AY751164 | BE/10843/00 |
| AY524616 | Ken/19/02 | AY751163 | BE/10701/00 |
| AY524615 | Ken/18/02 | AY751162 | BE/12353/01 |
| AY524614 | Ken/170/02 | AY751161 | BE/91/03 |
| AY524613 | Ken/17/02 | AY751160 | BE/1153/03 |
| AY524612 | Ken/17/01 | AY751159 | BE/1134/03 |
| AY524611 | Ken/169/02 | AY751158 | BE/15760/97 |
| AY524610 | Ken/168/02 | AY751157 | BE/12543/96 |
| AY524609 | Ken/165/02 | AY751156 | BE/854/98 |
| AY524608 | Ken/164/02 | AY751155 | BE/621/98 |
| AY524607 | Ken/163/02 | AY751154 | BE/13833/97 |
| AY524606 | Ken/162/02 | AY751153 | BE/73/98 |
| AY524605 | Ken/161/02 | AY751152 | BE/14819/97 |
| AY524604 | Ken/160/02 | AY751151 | BE/13436/02 |
| AY524603 | Ken/16/02 | AY751150 | BE/12894/99 |
| AY524602 | Ken/16/01 | AY751149 | BE/1950/01 |
| AY524601 | Ken/159/02 | AY751148 | BE/15116/98 |
| AY524600 | Ken/158/02 | AY751147 | BE/14517/97 |
| AY524599 | Ken/15/02 | AY751146 | BE/548/00 |
| AY524598 | Ken/15/01 | AY751145 | BE/382/00 |
| AY524597 | Ken/147/02 | AY751144 | BE/15111/98 |
| AY524596 | Ken/142/02 | AY751143 | BE/14607/98 |
| |AY524595 | Ken/14/02 | AY751142 | BE/9443/02 |
| AY524594 | Ken/14/01 | AY751141 | BE/12953/99 |
| AY524593 | Ken/130/02 | AY751140 | BE/14548/98 |
| AY524592 | Ken/13/02 | AY751139 | BE/351/00 |
| AY524591 | Ken/13/01 | AY751138 | BE/22/00 |
| AY524590 | Ken/128/02 | AY751137 | BE/12860/99 |
| AY524589 | Ken/123/02 | AY751136 | BE/14580/98 |
| AY524588 | Ken/12/02 | AY751135 | BE/14522/98 |
| AY524587 | Ken/12/01 | AY751134 | BE/12321/98 |
| AY524586 | Ken/119/02 | AY751133 | BE/12726/98 |
| AY524585 | Ken/118/02 | AY751132 | BE/12015/96 |
| AY524584 | Ken/11/02 | AY751131 | BE/13417/99 |
| AY524583 | Ken/11/01 | AY751130 | BE/12369/01 |
| AY524582 | Ken/10/02 | AY751129 | BE/11683/01 |
| AY524581 | Ken/10/01 | AY751128 | BE/11702/01 |
| AY524580 | Ken/1/02 | AY751127 | BE/758/02 |
| AY524579 | Ken/1/01 | AY751126 | BE/11535/01 |
| AY773286 | Ken/177/02 | AY751125 | BE/12446/01 |
| AY773287 | Ken/176/02 | AY751124 | BE/302/04 |
| AY773288 | Ken/232/02 | AY751123 | BE/1066/03 |
| AY773290 | Ken/202/02 | AY751122 | BE/553/03 |
| AY773293 | Ken/235/02 | AY751121 | BE/12349/02 |
| AY773294 | Ken/207/02 | AY751120 | BE/12394/02 |
| AY773295 | Ken/200/02 | AY751119 | BE/12522/01 |
| AY773296 | Ken/199/02 | AY751118 | BE/12370/01 |
| AY773297 | Ken/234/02 | AY751117 | BE/13159/02 |
| AY773298 | Ken/233/02 | AY751116 | BE/11508/01 |
| AY773299 | Ken/196/02 | AY751115 | BE/626/02 |
| AY773300 | Ken/183/02 | AY751114 | BE/13242/02 |
| AY773301 | Ken/184/02 | AY751113 | BE/12158/03 |
| AY728170 | Zhejiang/04/004 | AY751112 | BE/11732/01 |
| AY728169 | Zhejiang/04/003 | AY751111 | BE/210/03 |
| AY728168 | Zhejiang/04/002 | AY751110 | BE/46/03 |
| AY728167 | Zhejiang/04/001 | AY751109 | BE/1802/03 |
| AY472102 | sal/54/99 | AY751108 | BE/12598/01 |
| AY472097 | sal/74/99 | AY751107 | BE/12595/01 |
| AY472096 | sal/108/99 | AY751106 | BE/12379/01 |
| AY472095 | sal/81/99 | AY751105 | BE/11500/01 |
| AY472094 | sal/173/99 | AY751104 | BE/1162/02 |
| AY472093 | sal/82/99 | AY751103 | BE/1613/02 |
| AY472092 | sal/138/99 | AY751102 | BE/11609/01 |
| AY472091 | Sal/154/99 | AY751101 | BE/1584/02 |
| AY472090 | Sal/151/99 | AY751100 | BE/11813/01 |
| AY472089 | sal/136/99 | AY751099 | BE/12653/01 |
| AY472088 | sal/149/99 | AY751097 | BE/11949/01 |
| AY472087 | sal/140/99 | AY751096 | BE/12398/01 |
| AY472086 | sal/87/99 | AY751095 | BE/1192/02 |
| AB175818 | NG-040-03 | AY751094 | BE/12445/99 |
| AB175817 | NG-065-02 | AY751093 | BE/14610/03 |
| AB175816 | NG-042-02 | AY751092 | BE/257/04 |
| AB175815 | NG-009-02 | AY751091 | BE/12817/03 |
| AB175814 | NG-001-02 | AY751090 | BE/788/04 |
| AF065258 | CH57 | AY751089 | BE/12973/03 |
| AF065257 | CH3493_94 | AY751088 | BE/13058/03 |
| AF065256 | CH28 | AY751087 | BE/13457/03 |
| AF065255 | CH17 | AY751086 | BE/920/03 |
| AF065254 | CH09 | AY751085 | BE/920/03 |
| AF233923 | TX69564 | AY751084 | BE/12358/02 |
| AF233922 | TX69343 | AF309684 | Moz/204/99 |
| AF233921 | TX68532 | AF309683 | Moz/267/99 |
| AF233920 | TX68841 | AF309682 | Moz/46/99 |
| AF233919 | TX67951 | AF309681 | Moz/26/99 |
| AF233918 | NY20 | AF309680 | Moz/197/99 |
| AF233917 | NY108 | AF309679 | Moz/211/99 |
| AF233916 | NY103 | AF309678 | Moz/205/99 |
| AF233915 | MO55 | AF309677 | Moz/202/99 |
| AF233914 | MO48 | AF309676 | Moz/198/99 |
| AF233913 | MO16 | AF309675 | Moz/41/99 |
| AF233912 | MO15 | AF309674 | Moz/28/99 |
| AF233911 | MO13 | AF309673 | Moz/43/99 |
| AF233910 | MO02 | AF309672 | Moz/29/99 |
| AF233909 | MO01 | AF309671 | Moz/25/99 |
| AF233908 | CN3114 | AF309670 | Moz/50/99 |
| AF233907 | CN2851 | AF30969 | Moz/44/99 |
| AF233906 | CN2708 | AF309668 | Moz/47/99 |
| AF233905 | CN2395 | AF309667 | Moz/48/99 |
| AF233904 | CN1973 | AF309666 | Moz/14/99 |
| AF233903 | AL19556-3 | AF309665 | Moz/11/99 |
| AF233902 | AL19471-5 | AY927413 | QUE/191/01-02 |
| AF233901 | AL19452-2 | AY927412 | QUE/155/01-02 |
| AF233900 | AL19376-1 | AY927411 | QUE/146/01-02 |
| AF065410 | WV23836 | AY927410 | QUE/108/01-02 |
| AF065409 | WV12342 | AY927409 | QUE/99/01-02 |
| AF065408 | WV19983-87 | AY927408 | QUE/85/02-03 |
| AF065407 | WV6973-82 | AY927407 | QUE/73/02-03 |
| AF065406 | WV5222 | AY927406 | QUE/70/01-02 |
| AF065405 | WV2780-79 | AY927405 | QUE/54/01-02 |
| AF193327 | SK98579 | AY927404 | QUE/50/02-03 |
| AF193326 | SK98523 | AY927403 | QUE/43/02-03 |
| AF193325 | SK98072 | AY927402 | QUE/29/01-02 |
| AF193324 | SK97434 | AY927401 | QUE/18/02-03 |
| AF193323 | SK97385 | DQ289649 | BeijingB/04/11 |
| AF193322 | SK96308 | DQ289648 | Beijing/B/04/66 |
| AF193321 | SK96113 | DQ227408 | BA/1526/04 |
| AF193320 | SK95107 | DQ227407 | BA/493/04 |
| AF193319 | SK95118 | DQ227406 | BA/354/04 |
| AF193318 | SK95103 | DQ227405 | BA/5021/03 |
| AF193317 | SK95026 | DQ227404 | BA/4909/03 |
| AF193316 | SK94191 | DQ227403 | BA/4852/03 |
| AF193315 | SK94118 | DQ227402 | BA/4830/03 |
| AF193314 | SK93057 | DQ227401 | BA/4825/03 |
| AF193313 | SK93006 | DQ227400 | BA/693/03 |
| AF193312 | SK92528 | DQ227398 | BA/1326/99 |
| AF193311 | SK92415 | DQ227397 | BA/1607/04 |
| AF193310 | SK92359 | DQ227396 | BA/524/04 |
| AF193309 | SK92308 | DQ227395 | BA/100/04 |
| AF193308 | SK92011 | DQ227394 | BA/6564/03 |
| AF193307 | SK91399 | DQ227393 | BA/5140/03 |
| AF193306 | SK91242 | DQ227392 | BA/4974/03 |
| AF193305 | SK91142 | DQ227391 | BA/4862/03 |
| AF193304 | SK91087 | DQ227390 | BA/4915/03 |
| AJ290221 | UK70160/02/96 | DQ227389 | BA/4826/03 |
| AJ290220 | UK70161/02/96 | DQ227388 | BA/1889/02 |
| AJ290218 | UK71492/02/96 | DQ227387 | BA/1856/02 |
| AJ290217 | UK71128/01/96 | DQ227386 | BA/1606/02 |
| AJ290212 | UK70090/01/96 | DQ227385 | BA/1565/02 |
| AJ290208 | UK71360/12/95 | DQ227384 | BA/1562/02 |
| AJ290209 | UK70457/12/95 | DQ227383 | BA/1518/02 |
| X73354.1 | RSB642_1989 | DQ227382 | BA/1461/02 |
| X73353.1 | RSB6256_1989 | DQ227381 | BA/1441/02 |
| X73352.1 | RSB6190_1989 | DQ227380 | BA/1271/02 |
| X73351.1 | RSB5857_1989 | DQ227379 | BA/1214/02 |
| X73350.1 | RSB1734_1989 | DQ227378 | BA/1208/02 |
| X73355.1 | RSB6614_1989 | DQ227377 | BA/1161/02 |
| DQ289646.1 | Changchun/A/91/05 | DQ227376 | BA/1004/02 |
| DQ289645.1 | Changchun/A/91/04 | DQ227375 | BA/998/02 |
| DQ289644.1 | Changchun/A/91/02 | DQ227374 | BA/733/02 |
| DQ289643.1 | Changchun/A/91/01 | DQ227373 | BA/770/02 |
| DQ289642.1 | Lanzhou/A/04/01 | DQ227372 | BA/619/02 |
| DQ289641.1 | Beijing/A/04/62 | DQ227371 | BA/505/02 |
| DQ289640.1 | Beijing/A/04/57 | DQ227370 | BA/495/02 |
| DQ289639.1 | Beijing/A/04/56 | DQ227369 | BA/167/02 |
| DQ289638.1 | Beijing/A/04/54 | DQ227368 | BA/166/02 |
| DQ289637.1 | Beijing/A/04/53 | DQ227367 | BA/164/02 |
| DQ289636.1 | Beijing/A/04/51 | DQ227366 | BA/3997/99 |
| DQ289635.1 | Beijing/A/04/50 | DQ227365 | BA/3931/99 |
| DQ289634.1 | Beijing/A/04/48 | DQ227364 | BA/1370/99 |
| DQ289633.1 | Beijing/A/04/58 | DQ227363 | BA/802/99 |
| DQ289632.1 | Beijing/A/04/44 | AY333364 | BA4128/99B |
| DQ289631.1 | Beijing/A/04/43 | AY333363 | BA3859/99B |
| DQ289630.1 | Beijing/A/04/42 | AY333362 | BA3833/99B |
| DQ289629.1 | Beijing/A/04/41 | AY672701 | BA/5997/01 |
| DQ289628.1 | Beijing/A/04/39 | AY672700 | BA/5954/01 |
| DQ289627.1 | Beijing/A/04/38 | AY672699 | BA/4062/99 |
| DQ289626.1 | Beijing/A/04/37 | AY672698 | BA/3976/99 |
| DQ289625.1 | Beijing/A/04/36 | AY672697 | BA/3923/99 |
| DQ289624.1 | Beijing/A/04/35 | AY672696 | BA/3910/99 |
| DQ289623.1 | Beijing/A/04/34 | AY672695 | BA/3835/99 |
| DQ289622.1 | Beijing/A/04/33 | AY672694 | BA/3773/99 |
| DQ289621.1 | Beijing/A/04/32 | AY672693 | BA/3768/99 |
| DQ289620.1 | Beijing/A/04/31 | AY672692 | BA/3737/99 |
| DQ289619.1 | Beijing/A/04/52 | AY672691 | BA/3018/98 |
| DQ289618.1 | Beijing/A/04/27 | AY672690 | BA/2960/98 |
| DQ289617.1 | Beijing/A/04/28 | AY672689 | BA/2935/98 |
| DQ289616.1 | Beijing/A/04/23 | AY672688 | BA/2574/97 |
| DQ289615.1 | Beijing/A/04/22 | AY672687 | BA/2560/97 |
| DQ289614.1 | Beijing/A/04/21 | AY672686 | BA/1685/95 |
| DQ289613.1 | Beijing/A/04/26 | AY672685 | BA/1682/95 |
| DQ289612.1 | Beijing/A/04/17 | AY333361 | Mon/15/90 |
| DQ289611.1 | Beijing/A/04/16 | AF251556 | 40745 |
| DQ289610.1 | Beijing/A/04/15 | AF249877 | 35017 |
| DQ289609.1 | Beijing/A/04/12 | AF248642 | 34895 |
| DQ289608.1 | Beijing/A/04/10 |  |  |
| DQ289607.1 | Beijing/A/04/09 |  |  |
| DQ289606.1 | Beijing/A/04/08 |  |  |
| DQ289605.1 | Beijing/A/04/07 |  |  |
| DQ289604.1 | Beijing/A/04/06 |  |  |
| DQ289603.1 | Beijing/A/04/05 |  |  |
| DQ289602.1 | Beijing/A/04/04 |  |  |
| DQ289601.1 | Beijing/A/04/02 |  |  |
| DQ289600.1 | Beijing/A/04/01 |  |  |
| DQ289599.1 | Beijing/A/01/10 |  |  |
| DQ289598.1 | Beijing/A/01/05 |  |  |
| DQ289597.1 | Beijing/A/01/01 |  |  |
| AY667096 | BA/6122/01 |  |  |
| AY667095 | BA/6091/01 |  |  |
| AY667094 | BA/5952/01 |  |  |
| AY667093 | BA/5948/01 |  |  |
| AY667092 | BA/5081/00 |  |  |
| AY667091 | BA/5066/00 |  |  |
| AY667090 | BA/5049/00 |  |  |
| AY667089 | BA/5046/00 |  |  |
| AY667088 | BA/5017/00 |  |  |
| AY667087 | BA/4975/00 |  |  |
| AY667086 | BA/4940/00 |  |  |
| AY667085 | BA/4916/00 |  |  |
| AY667084 | BA/3961/99 |  |  |
| AY667083 | BA/3892/99 |  |  |
| AY667082 | BA/3858/99 |  |  |
| AY667081 | BA/3793/99 |  |  |
| AY667080 | BA/3771/99 |  |  |
| AY667079 | BA/3144/98 |  |  |
| AY667078 | BA/2964/98 |  |  |
| AY667077 | BA/2961/98 |  |  |
| AY667076 | BA/2955/98 |  |  |
| AY667075 | BA/2902/98 |  |  |
| AY667074 | BA/2606/97 |  |  |
| AY667073 | BA/2240/96 |  |  |
| AY667072 | BA/2206/96 |  |  |
| AY667071 | BA/2155/96 |  |  |
| AY667070 | BA/2151/96 |  |  |
| AY667069 | BA/1704/95 |  |  |
| AY667068 | BA/1694/95 |  |  |
| AY927387 | QUE/191/02-03 |  |  |
| AY927386 | QUE/161/01-02 |  |  |
| AY927385 | QUE/129/02-03 |  |  |
| AY927384 | QUE/84/02-03 |  |  |
| AY927383 | QUE/78/01-02 |  |  |
| AY927382 | QUE/75/02-03 |  |  |
| AF30964 | Moz/201/99 |  |  |
| AF30963 | Moz/170/99 |  |  |
| AF30962 | Moz/169/99 |  |  |
| AF30961 | Moz/27/99 |  |  |
| AF30960 | Moz/167/99 |  |  |
| AF30959 | Moz/40/99 |  |  |
| AF30958 | Moz/33/99 |  |  |
| AF30957 | Moz/24/99 |  |  |
| AF30956 | Moz/12/99 |  |  |
| AF30955 | Moz/9/99 |  |  |
| AY910822 | ARG104-04 |  |  |
| AY910821 | ARG44-04 |  |  |
| AY910820 | ARG14-04 |  |  |
| AY910819 | ARG6885-03 |  |  |
| AY910818 | ARG5982-03 |  |  |
| AY910817 | ARG5541-03 |  |  |
| AY910816 | ARG5632-03 |  |  |
| AY910815 | ARG5057-03 |  |  |
| AY910814 | ARG4852-03 |  |  |
| AY910813 | ARG4385-03 |  |  |
| AY910812 | ARG4009-03 |  |  |
| AY910811 | ARG3846-03 |  |  |
| AY910810 | ARG1711-02 |  |  |
| AY910809 | ARG1692-02 |  |  |
| AY910808 | ARG1628-02 |  |  |
| AY910807 | ARG1541-02 |  |  |
| AY910806 | ARG1296-02 |  |  |
| AY910805 | ARG1141-02 |  |  |
| AY910804 | ARG958-02 |  |  |
| AY910803 | ARG753-02 |  |  |
| AY910802 | ARG180-02 |  |  |
| AY910801 | ARG11493-02 |  |  |
| AY910800 | ARG6209_01 |  |  |
| AY910799 | ARG6035_01 |  |  |
| AY910798 | ARG5823_01 |  |  |
| AY910797 | ARG5555_01 |  |  |
| AY910796 | ARG5384_01 |  |  |
| AY910795 | ARG6177_01 |  |  |
| AY910794 | ARG729_00 |  |  |
| AY910793 | ARG5666_01 |  |  |
| AY910792 | ARG650-00 |  |  |
| AY910791 | ARG577_00 |  |  |
| AY910790 | ARG545_00 |  |  |
| AY910789 | ARG536_00 |  |  |
| AY910788 | ARG392_00 |  |  |
| AY910787 | ARG251_00 |  |  |
| AY910786 | ARG248_00 |  |  |
| AY910785 | ARG228_00 |  |  |
| AY910784 | ARG123_00 |  |  |
| AY910783 | ARG 2656_99 |  |  |
| AY910782 | ARG2356_99 |  |  |
| AY910781 | ARG2269_99 |  |  |
| AY910780 | ARG2089_99 |  |  |
| AY910779 | ARG1652_99 |  |  |
| AY910778 | ARG1493_99 |  |  |
| AY910777 | ARG1452_99 |  |  |
| AY910776 | ARG1432_99 |  |  |
| AY910775 | ARG1324_99 |  |  |
| AY910774 | ARG505_99 |  |  |
| AY910773 | ARG347_99 |  |  |
| AY910772 | ARG22516_97 |  |  |
| AY910771 | ARG22843_97 |  |  |
| AY910770 | ARG22518_97 |  |  |
| AY910769 | ARG22514_97 |  |  |
